# The Opposite Roles of White Light in Regulating Germination of Fresh and Aged Seed in Tobacco

**DOI:** 10.3390/plants10112457

**Published:** 2021-11-14

**Authors:** Yao Wang, Min Zhang, Shuai Dong, Yi-Ling Liu, Zhen-Hua Li

**Affiliations:** 1 College of Agriculture, University of Guizhou, Guiyang 550025, China; gs.yaowang3@gzu.edu.cn (Y.W.); gs.minzhang21@gzu.edu.cn (M.Z.); gs.dongs20@gzu.edu.cn (S.D.); 2 Key Laboratory of Plant Resource Conservation and Germplasm Innovation in Mountainous Region (Ministry of Education), University of Guizhou, Guiyang 550025, China; ylliu1@gzu.edu.cn

**Keywords:** germination, light, fresh seed, aged seed, hormone sensitivity, hormone level, hormone signal

## Abstract

Light is one of the important environmental factors for seeds to evaluate whether the natural environment is appropriate for germination and subsequent seedlings emergence. The mechanism of light-mediated germination is mainly concerned with fresh seeds (FS) of model plants but is poorly understood in aged seeds. Here, the effects of light on germination of FS and naturally aged seeds (NAS) in tobacco and their relationship with plant hormones gibberellins (GA) and abscisic acid (ABA) were investigated. The results demonstrated that light promoted and inhibited the germination of FS and NAS, respectively. GA and ABA were involved in the germination control of NAS, as well as in FS. However, light suppressed GA signal and stimulated ABA signal in NAS, whereas it stimulated GA signal and suppressed ABA signal in FS. In addition, light stimulated the GA accumulation and reduction in ABA in FS while inhibiting the increase in GA level in NAS. Together, the present study demonstrates that light has opposite effects on the germination of FS and NAS, which are closely related to the metabolism and/or signaling of plant hormones ABA and GA.

## 1. Introduction

Seed dormancy can be considered as the inability of viable seeds to germinate under favorable conditions [1]. Germination begins with the uptake of water by dry seed and ends with the protrusion of their radicles [2,3]. The essential role of the plant hormones abscisic acid (ABA) and gibberellin (GA) in the control of dormancy and germination has been recognized in photophilic, photoneutral, and photophobic seeds [4,5,6,7,8]. ABA is a positive regulator of dormancy induction and maintenance and acts as a negative regulator of germination. GA releases dormancy and promotes germination by antagonizing ABA actions [9,10,11]. Genetic evidence suggests that seed germination is not only regulated by hormone levels but also by hormone signals. ABA-deficient mutants, such as *aba1-1* and ABA-insensitive mutants, such as *abi3,* show reduced seed dormancy [12,13,14]. In contrast, GA-deficient mutants such as *ga1-3* and GA-insensitive mutants such as *gid1-receptor triple*, *sly1-10,* and *gai* show impaired seed germination [15,16,17].

Light is an important environmental signal that regulates seed germination of small-seeded plants, such as *Arabidopsis*, lettuce, and tomato (*Solanum lycopersicum*), and it controls seed germination by affecting biosynthesis and/or signals ABA/GA [18,19]. In *Arabidopsis*, PIF1 (PHYTOCHROME-INTERACTING FACTOR 1) strongly inhibits PHYA- and (or) PHYB-induced germination under dark [20]. On the one hand, PIF1 promotes the transcription of the genes (*GA2ox2*, *ABA DEFICIENT1* (*ABA1*) and *NCED6*, *9*) and inhibits the expression of genes (*GA3ox1* and *CYP707A2*), leading to a low GA/ABA ratio and consequently inhibiting seed germination [21,22,23]. On the other hand, PIF1 also promotes the expression of genes (*ABI3* and *ABI5*), which positively regulate ABA signaling and genes (*RGA* and *GA-INSENSITIVE*) that negatively regulate GA signaling, thereby inhibiting seed germination [24,25,26]. Under light, PHYA and (or) PHYB are activated by light and subsequently promote the degradation of PIF1 [27,28], thereby releasing those genes controlled by PIF1, leading to an increase in the GA/ABA ratio and stimulating GA signaling for seed germination. In lettuce, light regulates GA biosynthesis and light- and GA-mediated regulation of ABA metabolism during germination of photoblastic lettuce seeds [29,30].

It is assumed that light has at least two effects on the germination of tobacco seeds [31]. First, it activates the PHY signal pathway to release photodormancy and promote germination. Second, light accelerates rupture of the endosperm in non-photodormant tobacco seeds. In tobacco, testa rupture and endosperm rupture are separate events during seed germination. ABA delays endosperm rupture but not testa rupture [32]. GA can substitute for the red-light trigger needed to release photodormancy and to induce both testa rupture and the subsequent endosperm rupture of tobacco seeds imbibed in the dark. GA also can promote the germination of non-photodormant tobacco seeds by counteracting the inhibitory effects of ABA on endosperm rupture [33]. Several specific target enzymes, such as XTHs (xyloglucan endotransglycosylases),areinduced by GA in the micropylar endosperm during seed germination and proposed to promote endosperm weakening [3].

In photophilic seeds, light determines the levels and signals of gibberellin (GA) and abscisic acid (ABA), which promote and inhibit germination, respectively. Although this effect has been known in fresh seeds (FS) of *Arabidopsis* (*Arabidopsis thaliana*) and lettuce (*Lactuca sativa*), the molecular basis of light-regulated germination in aged seeds is still poorly understood. With the storage of seeds, the germination ratio of seed batches gradually decreases, and G_50_ (50% germination) is used as the standard to judge the survival of a batch of seeds. In this study, we noticed that the germination of FS was strongly inhibited by the entire spectrum of visible light in tobacco, while germination of NAS (G_50_) was promoted by the light. Kinetic curve analysis of hormones and related genes revealed that NAS has similar regulatory components as in FS for germination but with opposite responses to light.

## 2. Results

### 2.1. FS and NAS Germinate Better under Light and Dark Conditions

**Figure 1:**
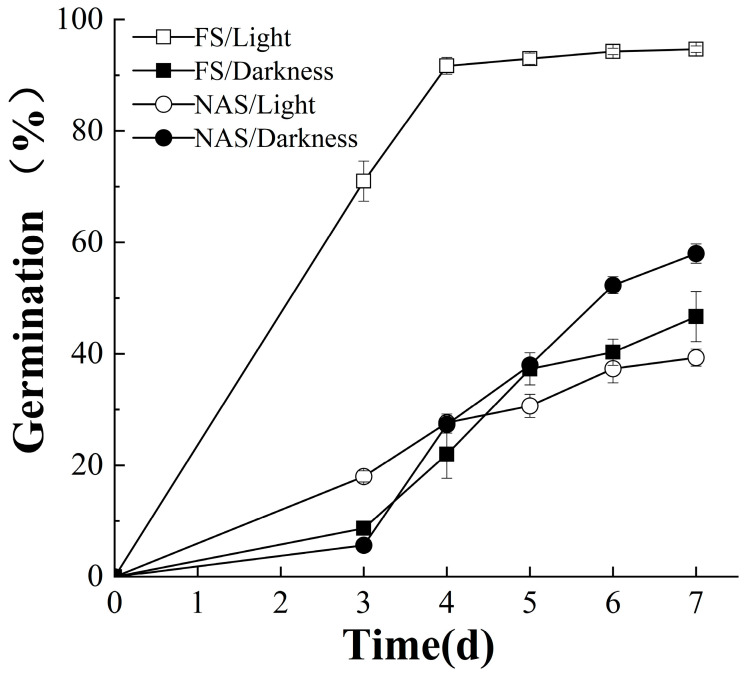
Light promotes and inhibits germination of freshly harvested seeds (FS) and naturally aged seeds (NAS), respectively. Percentage of germinating seeds kept in darkness (black graphics, the same Figs below) or under 12 h light/dark (white graphics, the same Figs below) cycle and 325 μmol m^−2^ s^−1^ white light condition, scored over time.

As shown in Figure 1, the germination ratio of FS was above 90% under light, which was significantly higher than that of 40% under dark. On the contrary, the germination ratio of NAS was higher under dark conditions and lower under light, with a difference of about 20%. The results indicated that the light promoted the germination of FS while inhibiting the germination of NAS.

### 2.2. GA Replaced Light to Promote and Inhibit Germination of FS and NAS

**Figure 2:**
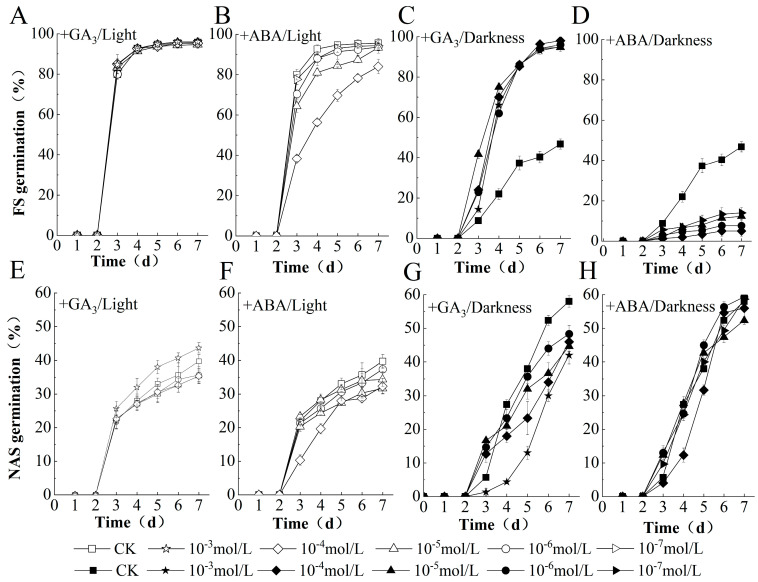
ABA and GA sensitivity of FS and NAS during germination. Percentage of germination on FS seeds (**A**–**D**) and NAS (**E**–**H**) kept in darkness or under 12 h light/dark cycle and 325 μmol m^−2^ s^−1^ white light condition, and (+) indicates that the gradient concentration of gibberellin or abscisic acid is used for seed treatment.

Under dark, the germination of FS was significantly improved by the application of exogenous GA_3_; however, the germination of NAS was inhibited by GA_3_ application, and the inhibiting effects were enhanced with an increase in its concentration. Under light, exogenous GA_3_ unaffected the germination of FS, and it promoted the germination of NAS at a high concentration of 10^−3^ mol/L (Figure 2).

The germination of FS was inhibited by ABA treatment either under light or dark. The higher the concentration, the more obvious the inhibition. The germination of NAS was inhibited by ABA treatment under light, while the germination was promoted at some concentrations by ABA under dark (Figure 2). The above results indicated that the FS was sensitive to ABA and GA during the germination, while the NAS seems to have lost their sensitivity to ABA and GA, or at least the sensitivity is much lower. The sensitivity of FS to ABA was not affected by light, while its sensitivity to GA was affected by light.

### 2.3. The Germination of FS Depending on Light to Promote GA Biosynthesis and ABA Reduction

**Figure 3:**
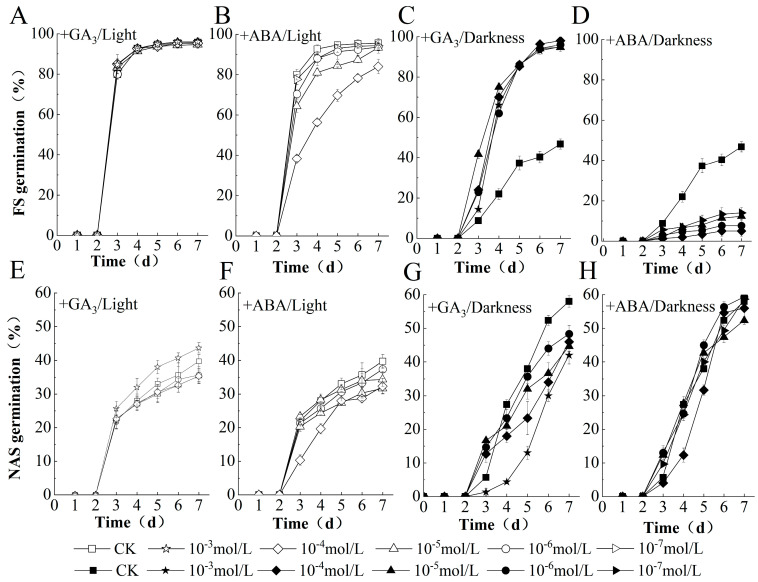
Kinetic curves of ABA and GA level in germinating FS and NAS. Hormone levels [in ng g^−1^ dry weight (DW)] of ABA (**A**), bioactive GA (**B**,**C**), and inactive GA (**D**–**G**) are shown during seed germination in tobacco. (**H**) Ratio of the averages from GAs (Sum of active and inactive GA) and ABA measurements.

In dry seeds, the ABA level in NAS was higher than that in FS (Figure 3A). After imbibition, ABA levels of NAS continued to decrease under light, while increased first and then subsequently decreased under dark. ABA levels of FS showed opposite changes, which continuously decreased and increased when they were incubated under light and dark, respectively. These results indicated that light promotes the decrease in ABA levels in both FS and NAS.

In dry seeds, GA_6_, GA_9,_ and GA_15_ levels were significantly higher in NAS than those in FS (Figure 3D–F), while GA_1_, GA_3,_ and GA_34_ levels showed no significant differences. (Figure 3B,C,G). After imbibition, the levels of GA_9_ and GA_15_ continued to decrease in NAS under both light and dark, reaching a level similar to those in FS. In both FS and NAS, the GA_34_ level was higher when the seeds were cultivated under light than under dark, or at least equal. The level of GA_6_ increased first and then decreased in FS or NAS, and its levels in FS showed that cultivated under light is lower than under dark, while showed higher under light in NAS. The changes of active GAs are similar in FS and NAS incubated under dark, with GA_1_ and GA_3_ showing a gradual increase after imbibition. Notably, the changes of active GAs are different in FS and NAS under light, with a significant increase in GA_1_ and GA_3_ on the third day in FS and not in NAS.

In dry seeds, the ABA/GA ratio in FS was significantly higher than that in NAS (Figure 3H). After imbibition, the ratios of ABA/GAs in both FS and NAS decreased quickly under light. Under dark, the ratio of ABA/GA in FS appears to decrease first and then increase, while ABA/GA ratio in NAS showed, increase first and then decrease. The ABA/GA ratios of both FS and NAS were lower under light than those under dark.

The above results indicate that the higher germination rate of FS under light conditions depends on the increase in active GA and the decrease in ABA level resulting in a lower ABA/GA ratio; while the lower germination under dark conditions is due to the decrease in active GA and the increase in ABA level leading to a higher ABA/GA ratio. However, there was no similar regulatory mechanism in NAS as FS.

### 2.4. The Effect of ABA Catabolism on Germination of FS Promoted by Light

**Figure 4:**
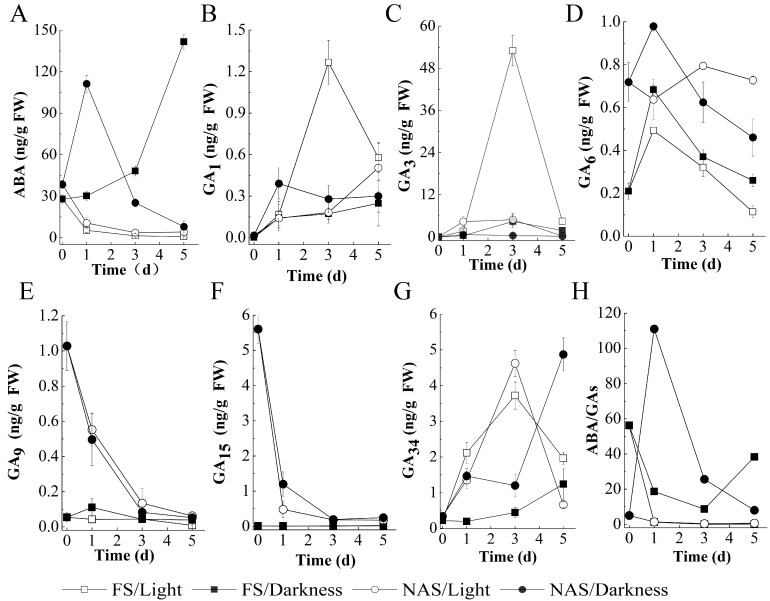
Expression levels of genes involved in ABA (**A**–**D**) or GA (**E**–**H**) metabolism or signal in FS or NAS during germination.

In *Arabidopsis*, *NCED6* is considered to be a key gene regulating ABA biosynthesis, whereas*CYP707A1* is involved in regulating ABA catabolic metabolism. No significant differences in the expression levels of *NtNCED6* and *NtCYP707A1* were observed between FS and NAS in the dry state (Figure 4A,B). After imbibition, the expression levels of *NtNCED6* were generally decreased either in FS or NAS under light and dark, but the decline speed of FS was significantly faster than that of NAS. The expression of *NtNCED6* was down- and upregulated by light in FS and NAS, respectively. Notably, *NtCYP707A1* was specifically highly expressed in FS on the first day, and its expression was significantly upregulated by light. These results indicate that ABA anabolism could be inhibited and stimulated by light in FS and NAS, respectively, while ABA catabolism could be stimulated by light in FS.

ABI3 and ABI5 are considered positive regulators of ABA signaling pathways, and they inhibit seed germination significantly in *Arabidopsis*. *NtABI5* and *NtABI3* were more highly expressed in the dry state of FS than that of NAS (Figure 4C,D). The expression level of *NtABI3* was quickly decreased in both FS and NAS on the first day, and light decelerated the decline speed. Notably, the *NtABI3* expression level specificity increased on the third day of dark culture in NAS. The expression of *NtABI5* showed a different trend, and its expression level in FS showed a continuous decrease, while in NAS, it showed an increase first and then a decrease. Light accelerated the decrease in the expression level of *NtABI5* in FS. These results suggest that the light-promoted germination of FS could be due to the reduction in ABA levels and suppression of its signal, while ABA anabolism and its signaling appear to be stimulated at the imbibition stage in NAS.

### 2.5. The Effect of GA Levels on Germination of FS Promoted by Light

*GA3ox* is the key regulatory gene for GA biosynthesis, whereas*GA2ox* mediates GA degradation. As shown in Figure 4E, the expression level of *NtGA3ox2* did not show a significant difference between NAS and FS. After imbibition, the expression of *NtGA3ox2* showed an opposite change between FS and NAS. The expression level of *NtGA3ox2* in FS increased first, then decreased, and then increased, while the expression level in NAS decreased first, followed by an increase and then a decline. The expression of *NtGA3ox2* in FS is upregulated by light while not in NAS.

*NtGA2ox2* expression is significantly higher in the dry state of NAS than that in FS (Figure 4F). The expression level of *NtGA2ox2* in NAS is decreasing both under light and dark. The expression level of *NtGA2ox2* in FS increased first, followed by a decline under light, which decreased first and then increased under dark. Light up-regulates the expression of *NtGA2ox2* in NAS. These results indicated that light-promoted FS germination might be due to inhibition of GA catabolism and promotion of GA anabolism, while GA catabolism seems to be stimulated in NAS.

In the dry state, the expression level of *NtGAI* in NAS was significantly higher than that in FS (Figure 4G). After imbibition, its expression level in NAS was significantly reduced, especially under dark. However, the expression level in FS was significantly increased and then reduced. These results indicated that the photo-inhibited NAS germination might be due to the inhibition of the GA signal.

### 2.6. The Effect of Cell Wall Hydrolysis on Light-Promoted and -Inhibited Germination of FS and NAS

*XTHs* are induced by GA to promote the germination of *Arabidopsis* seeds. As shown in Figure 4H, the expression level of *NtXTH2* was significantly higher in NAS than FS in the dry state. After imbibition, *NtXTH2* was specificity highly expressed on DAI24inFS under light. These results suggest that the light-promoted germination of FS could be due to the cell wall hydrolysis, while light-inhibited germination of NAS may be due to the partial inhibition of cell wall hydrolysis.

## 3. Discussion

Light is an important environmental signal. That is involved in regulating the germination of photosensitive seeds. The freshly harvested tobacco seeds are photodormant, their germination is strongly inhibited in the dark, and light can initiate the germination of photodormant seeds [34,35]. In other small-seeded species, such as lettuce [29,30,36,37] and *Arabidopsis*, light has also been shown to be necessary to initiate the germination of fresh seeds [38,39]. In contrast, seed germination of tomato and *Aethionema arabicum* are inhibited by light [8,40]. Overall, the germination of light-requiring (positive photoblastic) seeds requires light initiation, whereas light-inhibited (negative photoblastic) seeds need to avoid the participation of light signals. In this study, we noticed that light does promote the germination of FS in tobacco but partly inhibits the germination of NAS. To date, there is no relevant report on photo-inhibition of the germination of positive photoblastic seeds in the literature. We, therefore, speculate that light may provide suitable and unsuitable environments for germination of FS and NAS in tobacco, respectively.

ABA is the main plant hormone involved in inducing and maintaining seed dormancy and inhibiting germination, and GA is a key phytohormone that promotes seed germination by antagonizing ABA [9,33]. It has been reported that exogenous addition of GA and ABA promotes and inhibits seed germination, respectively. The effect of GA is more obvious under dark conditions, while the effect of ABA inhibiting seed germination is significant under both light and dark conditions [34]. This study also found that exogenous GA significantly promoted the germination of FS in the dark; however, the germination of NAS was inhibited under dark at the same concentration of GA application. In addition, the inhibitory effect of ABA on NAS is not obvious, especially in dark conditions. It has been reported that 10 or 100 μmol/L ABA strongly inhibited seed germination under light or dark [34,41]. No obvious inhibitory effect was observed in this study. We speculate that this difference could be the genotypes of the seeds or the different cultivation environments of the mother plants. In addition, the germination bed may also affect the results of the experiment. Agar germination beds were chosen instead of paper beds in this study.

It is well known that light regulates seed germination in *Arabidopsis* by integrating ABA and GA metabolism, including decreased ABA level and increased GA level [18,28]. Similarly, the germination of lettuce seeds also depends on light to regulate the anabolism of gibberellin and the catabolism of abscisic acid [29,30]. In this study, we noticed that light promoted the germination of FS in tobacco, which depended on the reduction in ABA levels and increase in GA levels. However, light also stimulated the reduction in ABA level in NAS but inhibited its germination. Recently, it has been illustrated that the same ABA/GA key regulatory components are required in light-inhibited and light-requiring germination in the Brassicaceae, but the difference between the expression of genes for key regulators upon light exposure [8]. Overall, light-regulated germination is related to hormone balance [8]. In this study, we noted that the germination ratio of FS was higher under light with a lower ABA/GA ratio, while the germination was lower under dark with a higher ABA/GA ratio. However, the germination ratio of NAS was higher under dark with a higher ABA/GA ratio, while the germination was lower under light with a lower ABA/GA ratio.

In *Arabidopsis thaliana* seeds, the expression of GA anabolic genes *GA3ox1* and *GA3ox2* are enhanced by light, whereas GA-inactivated gene *GA2ox2* is repressed, resulting in an increase in GA level [42,43,44,45]. In this study, the expression of *NtGA3ox2* is upregulated by light in FS whereas down-regulated in NAS. *NtGA2ox2* is down-regulated by light in FS, whereas itis upregulated in NAS. This indicates that light promotes the increase in GA levels in FS because it not only promotes GA biosynthesis but also inhibits GA catabolism. However, light promotes the catabolism of active GA in NAS. The expression of ABA biosynthesis genes ABA1, *NCED6* (*9-CIS-EPOXYCAROTENOID DIOXYGENASE 6*), and *NCED9* are repressed by light in *Arabidopsis* seed, while ABA catabolism gene *CYP707A2* is upregulated, resulting in a decrease in ABA level [21,24,45]. In lettuce seeds, the expression of *LsNCED2* and *LsNCED4* is repressed by light, whereas *LsABA8ox4* is upregulated [29]. In this study, the expression of *NtNCED6* was repressed and promoted when they were incubated under light in FS and NAS, respectively. *NtCYP707A1* was specifically highly expressed in FS, and its expression was significantly upregulated by light. This indicates that light promotes the decrease in ABA level in FS because it not only inhibits ABA biosynthesis but also promotes ABA catabolism. However, light inhibits the biosynthesis of active GA in NAS.

The expressions of *GAI* (*GIBBERELLIC ACID-IN-SENSITIVE,* a negative regulator of GA signal) [24] and *ABI3, ABI 5* (*ABSCISIC ACID-INSENSITIVE 3, 5*, positive regulators of ABA signal) [25,46] are stimulated to inhibit germination under dark in *Arabidopsis* seed. Furthermore, the expressions of genes required for cell wall loosening, such as *EXP* (*EXPANSIN*) and *XTH* (*XYLOGLUCANENDO-TRANSGLYCOSYLASE/HYDROLASE*), are repressed to inhibit seed germination under dark [25]. In this study, the expression of *NtGAI* was stimulated by light to inhibit the germination of NAS, and the expression of *NtABI5* and *NtXTH* was, respectively, repressed and stimulated by light to promote the germination of FS.

It has been shown that the timing of GA and ABA action is different during seed germination in *Arabidopsis* [47]. ABA could be involved in the regulation of almost all processes in germinating tobacco seeds, including imbibition, storage mobilization, endosperm rupture, and radicle protrusion, etc. [48]. GAs, although required for the completion of germination, are not directly involved in many processes taking place during germination in *Arabidopsis* seed, occurring at a stage coinciding with or very close to radicle emergence [49,50]. In this study, we noticed that the ABA level of NAS in a dry state was significantly higher than that of FS, and both decreased after imbibition under light. However, their ABA level showed an opposite trend after imbibition under dark, decreasing in NAS and increasing in FS.GA level only significantly improved when FS was exposed to light and at the stage coinciding with or very close to radicle emergence. Therefore, we speculated that the insufficient of GA level leaded to the lower germination of NAS under light, while the higher germination of NAS under dark was more likely due to the reduction in ABA.

## 4. Materials and Methods

### 4.1. Plant Materials

The FS of *Nicotiana tabacum* L. “K326” was obtained from Tobacco Research Institute in Guizhou Province. Seed aging was carried out under natural conditions (an average daily temperature of 15 °C, a daily difference of 6 °C, and an average daily relative humidity of 76%). In the initial stage, the germination of seeds was monitored every three months. When the germination rate began to decrease, the germination of seeds was monitored every month. At the 39th month, the germination ratio reached 50% (G_50_), and the seeds were taken as NAS in this study. FS and NAS (G_50_) were used for seed germination and hormone sensitivity testing. Two independent experiments were performed to support the germination pattern of NAS in response to light conditions. Samples on the 1st, 3rd, and 5th days of germination were used to determine the hormone levels and quantify the expression levels of their metabolism and signal genes.

### 4.2. Seed Germination and Hormone Sensitivity Test

The seeds were disinfected with 0.5% copper sulfate solution for 15 min, then rinsed with distilled water three times, and finally surface dried with filter papers. The sterilization solution containing 0.8% AGAR was poured into disposable Petri dishes to prepare as AGAR germinating beds. A total of 10^−6^, 10^−5^, 10^−4^, 10^−3^ and 0 mol/L of GA_3_ (Sigma, St.Louis, MO, USA), and 10^−7^, 10^−6^, 10^−5^, 10^−4,^ and 0 mol/L ABA (Sigma) solutions were prepared, respectively. Seeds were sown on AGAR germinating beds with or without supplementation of ABA or GA_3._ All Petri dishes were placed in artificial climate chambers with a temperature of 25 °C, light intensity of 18,000 Lux, relative humidity of 80%, and photoperiod of dark or 12 h light/dark cycle. Germination tests were performed on three replicates of 100 seeds. Seeds were checked for germination every day for a total of seven days. Germination was notarized as the length of the observed radicle approximately equal to the length of the seed.

### 4.3. ABA and GAs Quantification

Fresh tobacco seed materials collected on the 0, 1st, 3rd, and 5th days of germination were immediately frozen in liquid nitrogen and stored at −80 °C until needed. GAs and ABA levels were detected as Met Ware (http://www.metware.cn/) described. A total of 38 GAs were tested. In brief, each sample was ground (30 Hz, 1 min) into powder with a grinder (MM 400, Retsch). A total of 50 mg of the powder was weighed and then extracted with a mixed liquid of methanol: water: formic acid = 15:4:1 (v:v:v), containing an appropriate amount of internal standard substance. A total of 10 μL TEA and 10 μL BPTAB were added into the extraction solution, after 1 h of reaction at 90 °C, the mixture was then blown dry with nitrogen. The extract was reconstituted with 100 μL of 80% methanol-water solution, passed through a 0.22 μm PTFE filter membrane, and placed in a sample bottle for LC-MS/MS analysis. The data acquisition instrument system includes Ultra Performance Liquid Chromatography (UPLC, ExionLC™ AD) and tandem mass spectrometry (MS/MS, QTRAP^®^ 6500+). The liquid phase conditions include: (1) Chromatographic column: ACQUITY HSS T3 column (1.8 µm, 100 mm × 2.1 mm). (2) Mobile phase: Phase A, ultrapure water (adding 0.05% formic acid), and Phase B, acetonitrile (adding 0.05% formic acid). (3) Gradient elution program: 0 min A/B is 90:10 (V/V), 0.5 min A/B is 95:5 (V/V), 8.0 min A/B is 5:95 (V/V), 9.0 min A/B is 5:95 (V/V), 9.1 min A/B is 95:5 (V/V), and 12.0 min A/B is 95:5 (V/V). (4) Flow rate 0.35 mL/min; column temperature 40 °C; injection volume 2μL. The mass spectrometry conditions mainly include: Electrospray ionization temperature was 500 °C, mass spectrometry voltage was 4500 V, curtain gas was 35 psi, and the collision-activated dissociation parameter was set to medium. In Q-Trap 6500+, each ion pair is scanned based on the optimized declustering potential and coll energy.

### 4.4. RNA Extraction and Quantitative RT-PCR

Total RNA was extracted by using a TIANGEN RNA prep pure plant plus kit (Polysaccharides and Polyphenolics-rich). The concentration and purity of RNA were determined by using a NanoDrop-2000. Reverse transcription of the extracted RNA was performed by using TaKaRaPrimeScript™ II 1st strand cDNA synthesis kit. RT-PCR was performed by using TaKaRaTB Green^®^*Premix Ex Taq™* II (Tli RNaseH Plus), Bulk fluorescence quantification kit 20 µL reaction system as follow: 10 µL TB *Green Premix Ex Taq* (2×) (Tli RNaseH Plus), 0.4 µL ROX Reference Dye (50×), 2µL diluted to 40 ng/µL cDNA, 0.8μL of the upstream and downstream primers, respectively (Table 1), and make up the rest with water. The conditions of the RT-PCR reaction were 95 °C pre-denaturation for 0.5 min, then 95 °C for 5 s, 58 °C for 30 s and 40 cycles. The relative expression level of each gene was determined by using the Step One Plus real-time PCR instrument, and each sample was repeated three times. The relative expression level was calculated according to the method provided by Livak and Schmittgen [51]. The primers are as follows: *NtGA3ox2, NtGA2ox2, NtGAI, NtNCED6, NtCYP707A1, NtABI3, NtABI5, NtXTH2, NtTOC1, NtPHYB1, Actin* (*Tac9*).

## 5. Conclusions

Light is an important environmental signal that regulates seed dormancy and germination. It promoted and inhibited the germination of FS and NAS in tobacco, respectively. During germination, the FS was more sensitive to ABA and GA compared to NAS, and light would affect their sensitivity. Light promoted the germination of FS and inhibited that of NAS both by integrating metabolisms and/or signals of ABA and GA. First, a light-stimulated GA signal suppressed the ABA signal in FS, whereas it suppressed the GA signal and stimulated the ABA signal in NAS. Secondly, light promoted the increase in GA levels and the reduction in ABA levels in FS, whereas light only promoted the reduction in ABA levels in NAS. Together, the present study demonstrates that light has opposite effects on the germination of FS and NAS, which are closely related to the balance of hormones ABA and GA.

## Figures and Tables

**Table 1:** Real-time PCR primers used for genes expression analysis.

Gene Name	Forward Primer (5′–3′)	Reverse Primer (5′–3′)
*NtGA3ox2*	TGGAAAAACTAGCCGGAAGA	GCCCATTTCATATCGTCCTTAC
*NtGA2ox2*	TTGGAGGACCACCATTGAGT	CAAGCTGTCTTGATCCCCTTT
*NtGAI*	TCCACTAACAACAGATGCAACAACAAG	ACAGCTTCAGCACACGCCATT
*NtNCED6*	AGTTTCGGGTTGGTGGATGCTAC	CTGTAATACGGACGCTATACGGAAGAT
*NtCYP707A1*	GGTGATTCTGCTGGTGTTGTCTCT	GGGATATAGCTTAATGGGCAGA
*NtABI3*	GAGTATCAGACCATGGAATCTGC	TTCCATCGCGGAGAATTG
*NtABI5*	CGCAAAAGGCGACTAACAA	ACACATCAAGGGCAACTCAA
*NtXTH2*	GGCTAGTCACCACATCAAGTACCTCA	CACCTGAAGACCTGTCAAGAACAAGAT
*NtTOC1*	TGCTTCCACCACTGCTGCTCATA	TCCTGTCTGCCGTTCATTAGTTCCT
*NtPHYB1*	GTGTGATACTGTGGTTGAGAGTGTGA	TTGAGGAATGTCGGTAGCAGGATAATG
*Actin(Tac9)*	CCTGAGGTCCTTTTCCAACCA	GGATTCCGGCAGCTTCCATT
